# Uptake of Technetium-99m Sestamibi (99mTc-MIBI) as a Predictor of Fracture Risk in the Appendicular Skeleton of Multiple Myeloma Patients at a National Reference Center

**DOI:** 10.7759/cureus.105969

**Published:** 2026-03-27

**Authors:** Reyna Sarahi Bañuelos Balderas, Luis Miguel Linares González, David Antonio Arguelles Pérez, Monserrat G Bastidas Martínez, Carlos D Baranzini, Ana Luisa Galicia-Zamalloa

**Affiliations:** 1 Orthopedic Oncology, Instituto Nacional de Rehabilitación Luis Guillermo Ibarra Ibarra, Mexico City, MEX; 2 Nuclear Medicine, Instituto Nacional de Rehabilitación Luis Guillermo Ibarra Ibarra, Mexico City, MEX; 3 Orthopedics, Instituto Nacional de Rehabilitación Luis Guillermo Ibarra Ibarra, Mexico City, MEX; 4 Articular Reconstruction and Arthroscopic Surgery, Instituto Nacional de Rehabilitación Luis Guillermo Ibarra Ibarra, Mexico City, MEX

**Keywords:** 99mtc-mibi, fractures, multiple myeloma, nuclear medicine, oncology

## Abstract

Background and objective

Skeletal involvement in multiple myeloma frequently results in pathological fractures, which significantly increase morbidity and functional disability. Advanced imaging modalities such as PET/CT and whole-body MRI are considered reference techniques for disease assessment; however, their availability remains limited in many healthcare systems. The objective of this study is to evaluate the association between 99mTc-MIBI uptake and pathological fracture occurrence in the appendicular skeleton of patients with multiple myeloma and to determine whether radiotracer uptake and lesion morphology predict fracture timing.

Methods

A retrospective observational study was conducted, including patients with histologically confirmed multiple myeloma who underwent technetium-99m sestamibi (99mTc-MIBI) scintigraphy between 2014 and 2021. Radiotracer uptake was assessed qualitatively. Fractures were confirmed radiographically and classified as early (less than six months) or late (more than six months). Logistic regression analysis was performed to identify independent predictors.

Results

The cohort included 121 patients (median age 61.5 ± 12.5 years), including 69 men (57%) and 52 women (43%). Uptake of 99mTc-MIBI occurred most frequently in the humerus (31; 27.9%), axial skeleton (19; 17.1%), and femur (10; 9.0%). Pathological fractures occurred in 94 patients (77.7%), including 86 early fractures (71.1%) and eight late fractures (6.6%). In bivariate analysis, cortical erosion (χ² = 2.9; p = 0.023) and age >63 years (t = 2.4; p = 0.02) were associated with fracture. Multivariate logistic regression identified expansile lesions with cortical erosion as the strongest predictor of fracture (OR 2.1; 95% CI: 0.8-5.7).

Conclusions

99mTc-MIBI scintigraphy may represent a valuable adjunct imaging modality for the assessment of skeletal involvement in patients with multiple myeloma, particularly in resource-limited settings where advanced imaging techniques such as whole-body MRI or PET/CT are not readily accessible. Although cortical erosion continues to be the most reliable structural predictor of impending fracture, 99mTc-MIBI uptake offers complementary functional insight into the metabolic activity and tumor burden of myelomatous lesions. This functional information may be especially relevant in identifying lesions at higher risk of progression or early structural compromise, even before overt radiographic changes become evident. Therefore, 99mTc-MIBI could potentially contribute to a more comprehensive risk assessment when integrated with conventional imaging findings and clinical parameters. However, given the heterogeneity of disease presentation and the limited sample size of current studies, including the present analysis, these findings should be interpreted with caution. Future prospective studies with larger cohorts and standardized imaging protocols are warranted to further elucidate the prognostic value of 99mTc-MIBI uptake and to determine its potential role in fracture risk stratification and treatment decision-making algorithms.

## Introduction

Multiple myeloma is the second most common hematologic malignancy and is characterized by the clonal proliferation of malignant plasma cells within the bone marrow, leading to the production of monoclonal immunoglobulins and progressive skeletal destruction [1,2]. Osteolytic bone disease represents one of the hallmark manifestations of this condition and occurs in up to 80% of patients during the course of the disease. These lesions result from an imbalance between osteoclastic activation and inhibition of osteoblastic activity, ultimately leading to bone fragility, pathological fractures, hypercalcemia, and significant impairment in quality of life [2,3].

Globally, multiple myeloma represents a substantial healthcare burden. According to recent epidemiological estimates, thousands of new cases are diagnosed annually, with a considerable proportion of patients developing skeletal-related events during disease progression. In Mexico, several thousand cases have been reported in recent years, with bone involvement representing one of the most frequent and clinically relevant complications. Early identification of skeletal damage is therefore essential for risk stratification, treatment planning, and the prevention of pathological fractures [2,3].

Conventional skeletal surveys using plain radiography have historically been the standard imaging modality for the initial evaluation of bone involvement in multiple myeloma. However, this technique has important limitations, particularly in the early stages of the disease, as a significant degree of bone mineral loss is required before lytic lesions become radiographically detectable. As a result, sensitive imaging modalities have increasingly been incorporated into the diagnostic algorithm, including whole-body MRI, 18F-FDG PET/CT, and nuclear medicine techniques [3,4].

Among these, bone scintigraphy using technetium-99m sestamibi (99mTc-MIBI) has emerged as a potentially valuable diagnostic tool. This lipophilic radiotracer accumulates preferentially within mitochondria-rich malignant plasma cells, allowing the detection of metabolically active lesions and providing indirect information regarding tumor burden and disease activity. Previous studies have demonstrated that 99mTc-MIBI uptake correlates with bone marrow infiltration and monoclonal protein production, suggesting a potential role not only in disease detection but also in prognostic assessment [3,4].

Despite the recognized utility of imaging in the evaluation of myeloma-related bone disease, the relationship between radiotracer uptake patterns and the risk of subsequent pathological fracture remains insufficiently characterized. Identifying imaging features associated with structural instability could contribute to earlier detection of patients at increased risk of skeletal complications and facilitate timely orthopedic or oncologic intervention [3,4]. Therefore, the objective of the present study was to evaluate the association between 99mTc-MIBI uptake and pathological fracture occurrence in the appendicular skeleton of patients with multiple myeloma and to determine whether radiotracer uptake and lesion morphology predict fracture timing.

## Materials and methods

Study design and ethical approval

This retrospective observational study included human participants treated at the Instituto Nacional de Rehabilitación Luis Guillermo Ibarra Ibarra in Mexico City, Mexico. The study protocol was reviewed and approved by the institutional Research and Ethics Committee (INRLGII 92/23). Due to the retrospective nature of the study and anonymization of patient data, the requirement for informed consent was waived in accordance with institutional guidelines and the Declaration of Helsinki.

Patient population

Consecutive patients with histologically confirmed multiple myeloma who underwent 99mTc-MIBI scintigraphy between January 2014 and December 2021 were identified from institutional databases. Inclusion criteria included a histologically confirmed diagnosis of multiple myeloma, availability of 99mTc-MIBI scintigraphy, availability of baseline laboratory data including β2-microglobulin and albumin, and a minimum clinical and imaging follow-up of six months. Exclusion criteria included incomplete clinical or imaging records, absence of nuclear medicine imaging, prior history of pathological fracture at the evaluated site before scintigraphy, and exclusive axial skeleton involvement without appendicular lesions.

Imaging protocol and definition of 99mTc-MIBI uptake

All patients underwent whole-body planar scintigraphy following intravenous administration of 99mTc-MIBI (dose range: 740-925 MBq). Image acquisition was performed approximately 10-20 minutes post-injection using a dual-head gamma camera. Radiotracer uptake was assessed qualitatively by experienced nuclear medicine physicians and defined as focal uptake greater than the surrounding normal bone background. Uptake was recorded by anatomical location, with particular attention to the appendicular skeleton.

Lesion characterization and fracture assessment

Lesions were classified based on conventional radiography findings into expansile lytic lesions without cortical erosion and expansile lesions with cortical erosion. Pathological fractures were identified based on radiographic evidence and confirmed through clinical records. Fractures were categorized according to timing relative to the scintigraphy study: early fractures occurring within six months and late fractures occurring after six months.

Anatomical classification

Although the primary focus of the study was the appendicular skeleton, lesions in the axial skeleton were recorded when present. However, only appendicular lesions were included in the main analytical models evaluating fracture risk, given their greater biomechanical relevance for pathological fracture.

Disease staging

Disease stage was determined using the International Staging System (ISS) for multiple myeloma based on β2-microglobulin and albumin levels [5].

Statistical analysis

All statistical analyses were performed using IBM SPSS Statistics for Windows, Version 25.0 (Released 2017; IBM Corp., Armonk, NY, USA). Continuous variables were assessed for normality using the Kolmogorov-Smirnov test. Normally distributed variables are presented as mean ± SD, whereas non-normally distributed variables are reported as median and IQR. Categorical variables are expressed as absolute frequencies and percentages (n, %). Comparisons between groups were conducted according to the distribution and type of variable. Student’s t-test was used for normally distributed continuous variables, whereas the Mann-Whitney U test was applied for nonparametric data. Associations between categorical variables were evaluated using the chi-square (χ²) test or Fisher’s exact test, when appropriate.

To identify potential predictors of pathological fractures, a bivariate logistic regression analysis was initially performed. Variables with a p-value <0.10 in the bivariate analysis were subsequently included in a multivariate logistic regression model using the Wald method to control for potential confounding factors and identify independent predictors of fracture occurrence. The results of logistic regression models are presented as ORs with 95% CIs. Model goodness-of-fit was evaluated using the Hosmer-Lemeshow test. Multicollinearity among predictor variables was assessed using the variance inflation factor. All statistical tests were two-tailed, and statistical significance was defined as a p-value <0.05.

## Results

A total of 121 patients with histologically confirmed multiple myeloma were included in the final analysis. The median age of the cohort was 61.5 years (SD 12.5; range, 35-89 years). Among them, 69 patients were male (57.0%), and 52 were female (43.0%). The characteristics of the lesions were significantly associated with sex (Table 1). Female patients had a 1.94-fold higher risk (95% CI, 0.8-4.2; p = 0.06) of developing expansile lesions with cortical erosion compared with male patients.

**Table 1:** Association between sex and lesion characteristics

Sex	Expansile lytic lesion with cortical erosion, n = 73 (%)	Expansile lytic lesion without cortical erosion, n = 48 (%)	Total, n = 121 (%)
Female	36 (49.3%)	16 (33.3%)	52 (43%)
Male	37 (50.7%)	32 (66.7%)	69 (57%)

**Figure 1:**
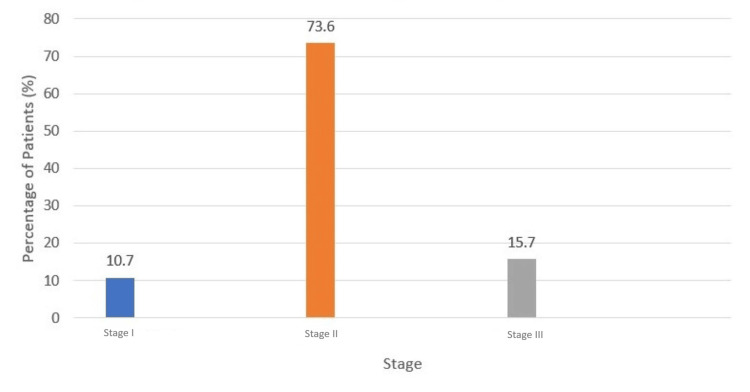
Distribution by stage in multiple myeloma

According to the ISS, most patients were classified as stage II, with a total of 89 patients (73.6%). Stage III disease was identified in 19 patients (15.7%), and stage I disease was observed in 13 patients (10.7%) (Figure 1). No missing data were identified for the variables included in the statistical analyses.

Distribution of 99mTc-MIBI uptake

**Figure 2:**
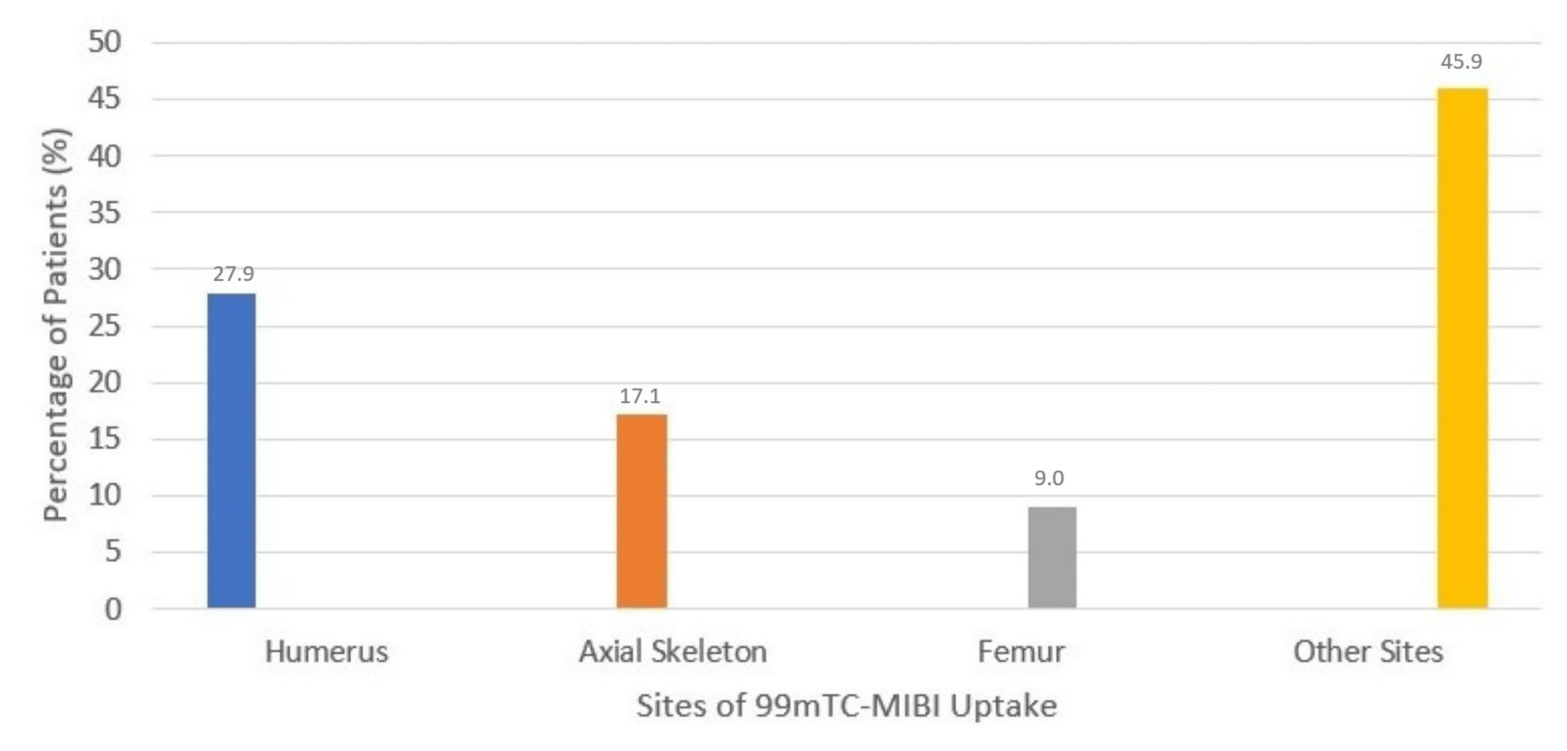
Distribution of 99mTc-MIBI uptake sites in the appendicular skeleton 99mTc-MIBI, technetium-99m sestamibi

The most frequent anatomical site of 99mTc-MIBI uptake in the appendicular skeleton was the humerus (31 patients; 27.9%), followed by the axial skeleton (19 patients; 17.1%), and the femur (10 patients; 9.0%). Other skeletal locations accounted for 51 cases (45.9%). Radiotracer uptake was not observed in 10 patients (8.3%) (Figure 2).

**Figure 3:**
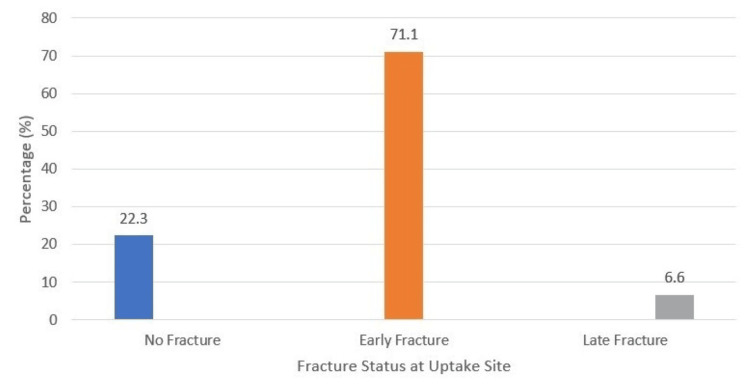
Fracture rate at the uptake site with 99mTc-MIBI 99mTc-MIBI, technetium-99m sestamibi

Fractures occurred in 94 patients (77.7%), including 86 early fractures (71.1%) and eight late fractures (6.6%) (Figure 3). The humerus was the most common site of uptake, observed in 31 patients (27.9%). Cortical erosion was present in nine patients with no fractures (13%), 54 patients with early fractures (78.4%), and five patients with late fractures (8.7%).

Bivariate analysis of factors associated with fracture

Age was significantly associated with fracture occurrence. Patients older than the cohort median (63 years) demonstrated a higher proportion of fractures compared with younger patients (t = 2.4; p = 0.02). Sex was not significantly associated with fracture occurrence (χ² = 2.1; p = 0.14). Among male patients, 50 individuals (72.5%) developed early fractures, whereas two patients (2.9%) developed late fractures. In contrast, 36 female patients (69.2%) developed early fractures, while six (11.5%) presented with late fractures.

Lesion morphology demonstrated a stronger association with fracture development. Among patients presenting with expansile lesions with cortical erosion, 60 cases (87%) developed fractures, whereas fractures occurred in 34 patients (75.6%) with expansile lytic lesions without cortical erosion. Although this difference did not reach statistical significance (χ² = 2.9; p = 0.23), the presence of cortical erosion showed a trend toward increased fracture risk. Several cases classified as late fractures initially presented as expansile lytic lesions without cortical erosion on imaging. Structural weakening progressed over time, with cortical erosion developing during disease evolution before fracture occurrence (Table 2).

**Table 2:** Factors associated with fracture at the uptake site

Factor	No fracture, n (%)	Early fracture, n (%)	Late fracture, n (%)	Statistic/p
Male	17 (24.6%)	50 (72.5%)	2 (2.9%)	χ² = 2.1; p = 0.14
Female	10 (19.2%)	36 (69.2%)	6 (11.5%)	
Expansile lytic lesion with cortical erosion	9 (13.0%)	54 (78.3%)	6 (8.7%)	χ² = 2.9; p = 0.23
Expansile lytic lesion without cortical erosion	11 (24.4%)	32 (71.1%)	2 (4.4%)	

Clinical stage according to the ISS was not significantly associated with fracture occurrence (χ² = 0.7; p = 0.42). Regarding laboratory markers, β2-microglobulin levels greater than 3 mg/dL showed no statistically significant association with fracture occurrence (χ² = 2.3; p = 0.13). Similarly, albumin and LDH levels were not significantly associated with fracture risk.

Logistic regression analysis

A logistic regression analysis was performed to identify independent predictors of pathological fracture. Variables with p < 0.10 in the bivariate analysis were included in the multivariate model. In the bivariate analysis, both 99mTc-MIBI uptake at appendicular skeletal sites and lesion morphology characterized by cortical erosion were evaluated as potential predictors of fracture. Uptake-positive lesions demonstrated a higher frequency of fractures compared with lesions without radiotracer uptake; however, this association did not reach statistical significance (χ² = 2.4; p = 0.11).

In the multivariate model, expansile lesions with cortical erosion emerged as the strongest imaging predictor of fracture, with an OR of 2.1 (95% CI, 0.8-5.7). Sex also showed a trend toward association with fracture risk. Male patients demonstrated a higher probability of fracture (OR = 3.3; 95% CI, 0.9-12.2; p = 0.06), although this did not reach statistical significance. Age greater than 63 years was independently associated with fracture risk (OR = 1.8; 95% CI, 0.9-3.4; p = 0.02) (Table 3). 99mTc-MIBI uptake did not remain an independent predictor of fracture in the multivariate model after adjustment for lesion morphology and demographic variables.

**Table 3:** Logistic regression analysis for predictors of early fracture

Variable	OR	95% CI	Wald	p-Value
Cortical erosion lesion	2.1	0.8-5.7	2.4	0.12
Male sex	3.3	0.9-12.2	3.5	0.06
Age >63 years	1.8	0.9-3.4	4.1	0.02

## Discussion

The principal finding of this study is that lesions characterized by cortical erosion are associated with a higher probability of pathological fracture, underscoring the critical role of structural bone compromise in the progression of multiple myeloma-related skeletal disease. Cortical disruption represents an advanced stage of osteolytic damage, reflecting the cumulative effects of increased osteoclastic activity and impaired osteoblastic bone formation, ultimately leading to mechanical instability and fracture susceptibility.

An important consideration arising from our findings is the complementary role of 99mTc-MIBI scintigraphy in this context. While cortical erosion can be readily identified using conventional radiography or computed tomography, these modalities primarily reflect established structural damage. In contrast, 99mTc-MIBI provides functional information by detecting metabolically active plasma cell infiltration. This distinction is clinically relevant, as radiotracer uptake may precede overt cortical disruption, allowing identification of biologically active lesions at an earlier stage of disease progression.

Bone involvement remains a hallmark of multiple myeloma, affecting approximately 70-80% of patients during the disease course. The underlying pathophysiology is driven by an imbalance between osteoclast activation and suppression of osteoblast-mediated bone formation, resulting in progressive osteolysis and weakening of the skeletal framework. Within this continuum, cortical erosion represents a pivotal event that significantly increases the likelihood of pathological fracture, as consistently reported in previous studies [1].

Advanced imaging modalities such as FDG-PET/CT and whole-body MRI are currently considered reference standards for disease staging and treatment monitoring. These techniques provide high sensitivity for detecting metabolically active lesions and assessing overall tumor burden, with important prognostic implications [6-8]. Nevertheless, their limited availability and high cost restrict their use in many healthcare systems, particularly in middle-income countries. In such settings, more accessible imaging alternatives remain essential.

In this context, 99mTc-MIBI scintigraphy represents a practical and widely available imaging modality. The radiotracer accumulates within mitochondria-rich plasma cells, and its uptake has been shown to correlate with tumor burden and metabolic activity [6-8]. As such, focal uptake may identify areas of active disease that are at risk of subsequent structural deterioration, even in the absence of radiographically evident cortical disruption.

An important clinical implication of our findings is that expansile lytic lesions without initial cortical erosion should not be considered biologically stable. Our data suggest that these lesions may represent an earlier stage of structural compromise, with the potential to progress toward cortical disruption and delayed pathological fracture. This observation supports the concept that metabolic activity, as detected by 99mTc-MIBI, may precede and potentially predict structural failure.

Furthermore, the high proportion of fractures occurring at sites of radiotracer uptake reinforces the potential value of scintigraphic patterns in risk stratification. Although 99mTc-MIBI uptake did not emerge as an independent predictor in multivariate analysis, its association with fracture occurrence suggests that it may still provide clinically relevant information when interpreted alongside structural imaging findings [9-11]. In this regard, 99mTc-MIBI should be considered a complementary tool rather than a standalone predictor.

This study has several methodological limitations that warrant consideration. First, its retrospective design introduces the possibility of selection bias and limits causal inference. Second, the sample size may have reduced the statistical power to detect significant associations, particularly in multivariate analyses. Third, the absence of systematic comparison with advanced imaging modalities such as PET/CT or whole-body MRI limits the ability to fully contextualize the diagnostic performance of 99mTc-MIBI. Some associations observed in this study, including the relationship between 99mTc-MIBI uptake and fracture risk, did not reach statistical significance and should therefore be interpreted with caution. These findings should be considered exploratory and hypothesis-generating rather than definitive.

Despite these limitations, the present study provides clinically relevant insights into the role of 99mTc-MIBI in the evaluation of skeletal involvement in multiple myeloma. In resource-limited settings, where access to advanced imaging is restricted, 99mTc-MIBI may serve as a useful adjunct modality for identifying metabolically active lesions and supporting early risk stratification of skeletal complications. Future prospective studies with larger sample sizes and direct comparison with advanced imaging techniques are needed to better define the role of 99mTc-MIBI within diagnostic and prognostic algorithms for multiple myeloma-related bone disease.

## Conclusions

99mTc-MIBI scintigraphy may represent a useful adjunct imaging modality for the evaluation of bone involvement in multiple myeloma, particularly in settings where advanced imaging techniques are not readily available. While cortical erosion remains the strongest structural predictor of fracture, 99mTc-MIBI uptake may provide complementary functional information regarding disease activity. Further prospective studies with larger sample sizes are required to validate its role in fracture risk stratification.
